# Does Parental Alcohol Use Influence Children’s Age at First Alcohol Intake? A Retrospective Study of Patients with Alcohol Dependence

**DOI:** 10.3390/healthcare9070841

**Published:** 2021-07-03

**Authors:** Dominika Berent, Marcin Wojnar

**Affiliations:** 1 Department of Psychiatry, Masovian Regional Psychiatric Hospital Drewnica, 05-091 Ząbki, Poland; 2 Department of Psychiatry, Medical University of Warsaw, 02-091 Warsaw, Poland; marcin.wojnar@wum.edu.pl; 3 Department of Psychiatry, University of Michigan, Ann Arbor, MI 48109, USA

**Keywords:** first alcohol intake, alcohol dependence, parental alcohol abuse/dependence

## Abstract

Parental alcohol misuse has detrimental effects on the entire family. In particular, the safety and general health of the children of parents with alcohol abuse/dependence are of concern to health authorities around the globe. The present study aimed to examine the impact of parental history of alcohol abuse/dependence on the age of first alcohol intake in adult patients with alcohol dependence. Questionnaire data were collected from 294 (57 females) patients with alcohol dependence (M ± SD, 42 ± 10.96 years). The majority of males (61.2%) and over half (50.9%) of females reported no history of parental alcohol abuse/dependence. Male patients with alcohol dependence were less likely to report living with both parents with alcohol abuse/dependence than female patients with alcohol abuse/dependence (*p* < 0.05). However, male patients who lived with both parents with alcohol abuse/dependence were younger at first alcohol intake than their female counterparts (median age: 12.00 vs. 18.00, *p* = 0.002) and males raised by parents without alcohol abuse/dependence (median age: 12.00 vs. 16.00, *p* = 0.036). Our findings suggest that age at first alcohol intake may serve as a marker of household dysfunction, including poor parental management. Our study supports the global need for systemic interventions to help alcohol abusing/dependent parents to carry out their parental responsibilities.

## 1. Introduction

According to the European Monitoring Centre for Drugs and Drug Addiction (2008), of almost 9,000,000 individuals under the age of 20 years surveyed in Poland in 2007, 17–23% reported at least one parent abusing alcohol or with alcohol dependence [1]. The safety and general health of children of parents with alcohol abuse/dependence are of concern to health authorities around the globe [2]. Children raised by one or more parents with alcohol abuse/dependence are at a higher risk for physical and non-physical abuse, neglect, and other consequences of poor parental practices, i.e., higher risk of injuries, earlier alcohol use, etc. [3,4,5,6,7,8,9]. The effects of maternal and paternal substance abuse (alcohol and illegal substances) on offspring may differ, as mothers often have the main responsibility of childcare [10]. Several studies have demonstrated that maternal substance abuse has more extensive adverse effects on the mental and physical well-being of their offspring than paternal substance abuse but that the effects are lower than if both parents are engaged in substance abuse [3,4]. The risk of injuries is found to be over two-fold higher among children of alcohol-abusing mothers and almost three-fold higher in families where both parents abuse alcohol, compared with the children of alcohol abstaining mothers [3]. Maternal substance abuse is shown to increase the risk of hospitalization for somatic illness (odds ratio (OR) 1.34) and psychiatric disorders (OR 1.33) in 0–6-year-old children, while paternal substance abuse increases the risk of hospitalization because of psychiatric disorders (OR 1.18) [9]. Notably, the risks are even higher if both parents are substance abusers [9]. Maternal alcohol use is found to be a significant independent risk factor for injury in children during the first year of life (OR 2.15) [4]. In young children (i.e., during the first few years of life), first alcohol intake is frequently considered to be an accident rather than a conscious intention to drink alcohol. However, this kind of accidental alcohol intake in early childhood is more prevalent among dysfunctional alcoholic families because of higher alcohol availability and poor management practices by parents [5,9]. Analysis of archival medical records of Polish pediatric units between the years 2000 and 2011 revealed that about 10% of mothers and over 20% of fathers of children admitted due to alcohol intoxication were diagnosed with alcohol dependence [11]. Only about 0.5% of mothers and 0.5% of father were found to be illegal substance users in the same study [11].

Living in a dysfunctional household, physical and non-physical abuse, and neglect have been linked to over 20 outcomes related to mental disturbances in adulthood, including alcohol dependence [12]. Adults with alcohol dependence tend to report a higher number of adverse childhood experiences than the general population [6,13,14]. Household substance abuse, which is defined as alcohol abuse/dependence or illegal drug use by any household member, is one of a number of adverse childhood experiences assessed in the Adverse Childhood Experiences Study, alongside other household dysfunction and childhood neglect and abuse present during the first 18 years of life [6,15]. Adults with alcohol dependence tend to report childhood household substance abuse more frequently than the general population [13,14]. In Poland, almost 47% of adult patients with alcohol dependence report childhood household substance abuse [13] vs. about 9% of the general population [14].

### Present Study

To our knowledge, this study is the first to assess the association between age at first alcohol intake and parental history of alcohol abuse/dependence in a sample of adult patients diagnosed with alcohol dependence. As mentioned above, maternal and paternal alcohol abuse/dependence may vary in their consequences for the health and well-being of offspring. Thus, we decided to separately assess the effects of mothers’, fathers’, or both parents’ alcohol abuse/dependence on age at first alcohol intake in offspring. Females are known to report higher prevalence rates of adverse childhood experiences, including childhood household substance abuse, in both clinical samples of individuals with alcohol dependence [13] and in the general population [6,14]. In Poland, childhood household substance abuse was self-reported by 42% of male and 60% of female adult patients with alcohol dependence [13]. Notably, males and females may develop different strategies to deal with experiences of childhood adversities, and females are twice as likely to develop trauma-related psychopathology compared to males [16,17]. Thus, we present our results separated by sex. We hypothesize that patients with alcohol dependence and any parental history of alcohol abuse/dependence are more likely to consume alcohol for the first time at an earlier age than their counterparts without parental history of alcohol abuse/dependence. Specifically, our aims were (1) to assess the prevalence of self-reported parental history of alcohol abuse/dependence among adult patients with alcohol dependence, and (2) to explore whether age at first alcohol intake differs with parental history of alcohol abuse/dependence.

## 2. Materials and Methods

### 2.1. Participants

This study included 294 (57 females) of 376 (80 females) initially enrolled patients with alcohol dependence. Eighty-two (23 females) patients were excluded from the analyses, due to meeting the study exclusion criteria detailed below. Participants provided informed consent and were informed in the consent form that they have the right to withdraw consent at any step of the study without giving any reason. Participants were ensured the confidentiality of the provided information. This study was approved by the local bioethics committee of our institutions: Nos. RNN/467/13/KB, KB/843/13/P, and AKBE/205/2018. The data were collected between 2013 and 2015 and in 2019. The study was carried out in accordance with the ethical standards laid down in the 1964 Declaration of Helsinki and its later amendments.

### 2.2. Procedures

Study participants were recruited from consecutive inpatients with alcohol dependence admitted to the psychiatric units of a hospital in central Poland. Patients with alcohol dependence were hospitalized for a course of psychotherapeutic treatment or for the treatment of alcohol withdrawal syndrome. The inclusion criteria were as follows: (1) age ≥ 18 years; (2) ≥1 week since last alcohol intake (to exclude patients with acute withdrawal syndrome at the time of the study); and (3) consensus diagnosis of alcohol dependence made by at least two psychiatrists according to the ICD-10 criteria, F10.2 [18]. The exclusion criteria were as follows (the number of individuals excluded due to each criterion is given in brackets): (1) previous diagnosis of another significant mental comorbidity, according to the ICD-10 criteria, in their lifetime based on available current and archival medical records (*n* = 11); (2) returning an incomplete questionnaire (*n* = 61); and (3) reporting another family member with alcohol abuse/dependence, other than the mother or father (*n* = 15). Note that some individuals met multiple exclusion criteria.

The researcher remained present during the completion of the study questionnaires to address participants’ questions and ensure that they understood all of the items. To minimize possible bias associated with participants’ intentional attempts to present him/herself in either a better or worse mental or general condition, the researcher who remained present during the completion of the questionnaires was not involved in the patients’ therapy.

### 2.3. Measures

#### 2.3.1. Demographic and Social Variables

Sociodemographic and clinical characteristics of the study participants (presented in Table 1) were assessed with a structured self-report questionnaire designed for this study.

#### 2.3.2. Alcohol Use Disorders Identification Test

The Alcohol Use Disorders Identification Test (AUDIT), which is an open-access tool, was used to characterize alcohol intake severity among patients with alcohol dependence. The sensitivity and specificity of the Polish version of AUDIT are 0.893 and 0.863, respectively [19].

#### 2.3.3. Age at First Alcohol Intake

Age at first alcohol intake (i.e., any amount of alcohol drank, including small tastes or sips) was assessed by the following question of the structured self-report questionnaire designed for this study: “How old were you when you first tasted alcohol?”

#### 2.3.4. Parental History of Alcohol Abuse/Dependence

Parental history of alcohol abuse/dependence was identified with the following question of the structured self-report questionnaire: “Prior to your 18th birthday, did you live with anyone who abused alcohol/was alcohol dependent? If yes, who was that?” Based on these data, patients were classified as (1) without parental history of alcohol abuse/dependence, (2) with maternal and paternal history of alcohol abuse/dependence, (3) with maternal history of alcohol abuse/dependence, or (4) with paternal history of alcohol abuse/dependence. Only patients without parental alcohol abuse/dependence or one or both parents’ alcohol abuse/dependence were analyzed in the study. As previously reported by Grant et al. (1994), family history items generally show good to excellent reliability, with kappa values of 0.72 for fathers’ and 1.00 for mothers’ history of alcohol abuse/dependence [20].

### 2.4. Statistical Analysis

Statistical analyses were performed using SPSS Statistics, version 25 (IBM Corp., USA). In the first step, descriptive statistics were analyzed, and the normality, skewness, and kurtosis were determined. Data are reported as the mean (M) or median (Me) ± standard deviation (SD). According to the Kolmogorov–Smirnov test with Lilliefors correction, the current age of female patients with alcohol dependence and AUDIT scores in female patients with alcohol dependence showed a normal distribution. For the remaining variables, the skewness values were in the range ± 2 (i.e., the deviation from a normal distribution was not significant), except for the females’ age at first alcohol intake [21]. To compare quantitative variables between two groups (i.e., both/none/any parents with alcohol abuse/dependence), the Mann–Whitney U test was used. Alternatively, the Kruskal–Wallis test was use for comparisons between more than two groups. To compare nominal variables, Fisher’s exact test was used. The level of statistical significance was set at a *p*-value < 0.05.

## 3. Results

### 3.1. Study Sample

The final study sample comprised 294 patients (57 females) with alcohol dependence aged 42 ± 10.96 (Me ± SD) years. The median AUDIT score was 28 ± 7.63 (Me ± SD) and median self-reported age at first alcohol intake was 16 ± 4.40 (Me ±S D) years (Table 1). Female patients with alcohol dependence were older at first alcohol intake (17.00 ± 5.98 (Me ± SD) vs. 16.00 ± 3.81 (Me ± SD); Z = −2.79; *p* = 0.005; r = 0.16), had lower AUDIT scores (22.00 ± 7.89 (Me ± SD) vs. 28.00 ± 7.27 (Me ± SD); Z = −4.05; *p* < 0.001; r = 0.24), and were less frequently never married (χ^2^(4) = 14.30; *p* = 0.006; V = 0.22), compared to male patients with alcohol dependence (Table 1).

The lowest self-reported age at first alcohol intake was 3 years for male patients with alcohol dependence and 10 years for female patients with alcohol dependence. For additional descriptive statistics regarding current age, age at alcohol initiation, and AUDIT scores in male and female patients with alcohol dependence, please see the Supplementary Materials Table S1. Age at alcohol initiation ≤ 14 years was self-reported by 10 (17.54%) female patients with alcohol dependence and 82 (34.60%) male patients with alcohol dependence.

The majority of male (61.2%) patients with alcohol dependence and over half of female (50.9%) patients with alcohol dependence reported living with parents without alcohol abuse/dependence. Significant differences were found when comparing male and female patients for the prevalence of living with alcohol abusing/dependent parent(s) (Table 1; *p* = 0.025; V = 0.19). Post-hoc analysis showed that the prevalence of living with both parents with alcohol abuse/dependence was lower among male patients with alcohol dependence, compared to female patients with alcohol dependence (*p* < 0.05).

### 3.2. Relationship of Age at First Alcohol Intake with Parental History of Alcohol Abuse/Dependence in Male and Female Patients with Alcohol Dependence

Male and female patients with alcohol dependence without history of alcohol abuse/dependence in both parents vs. those with maternal-only or paternal-only alcohol abuse/dependence did not differ in their age at first alcohol intake (Table 2; Z = −0.99, *p* = 0.323, r = 0.08; Z = 0.56, *p* = 0.616, r = 0.13; Z = − 1.82, *p* = 0.069, r = 0.20; respectively). Male patients raised by both parents with alcohol abuse/dependence were significantly younger at first alcohol intake than their female counterparts (Table 2; Z = −2.97, *p* = 0.002, r = 0.74).

### 3.3. Comparison of Age at First Alcohol Intake Among Male and Female Patients with Alcohol Dependence by Parental History of Alcohol Abuse/Dependence

There was a significant difference in age at first alcohol intake among male patients with different parental histories of alcohol abuse/dependence (Table 3; H = 10.10, *p* = 0.018, η^2^ = 0.04). Post hoc analysis showed that age at first alcohol intake among male patients was significantly lower in the subgroup that reported living with both parents with alcohol abuse/dependence compared to those who lived with parents without alcohol abuse/dependence (Table 3; *p* = 0.036).

There was no significant difference in age at first alcohol intake among female patients when comparing subgroups with different parental histories of alcohol abuse/dependence (Table 3; H = 3.37, *p* = 0.338, η^2^ = 0.06).

## 4. Discussion

The present study examined whether parental alcohol abuse/dependence was associated with age at first alcohol intake in adult patients with alcohol dependence. The majority of the male patients with alcohol dependence (61.2%) in our sample and over half (50.9%) of the female patients with alcohol dependence reported growing up without alcohol abusing/dependent parents. According to the parental history of alcohol abuse/dependence, our adult male and female patients with alcohol dependence were divided into subgroups: (1) those without history of parental alcohol abuse/dependence, (2) those with both parents with alcohol abuse/dependence, (3) those with maternal history of alcohol abuse/dependence, and (4) those with paternal history of alcohol abuse/dependence. In all subgroups, the female patients with alcohol dependence were older than their male counterparts at first alcohol intake. However, this sex difference only reached statistical significance between males and females who were raised by both alcohol abusing/dependent parents. Among female patients with alcohol dependence, there was no difference in age at first alcohol intake between subgroups. Among males, however, there was a significant difference in age at first alcohol intake such that male patients who were raised by both parents with alcohol abuse/dependence were younger at first alcohol intake than male patients raised by parents without alcohol abuse/dependence.

In our sample, male and female patients with alcohol dependence did not differ significantly in their current age. Thus, the time since childhood and adolescence did not differ between male and female patients. Male patients showed higher AUDIT scores than female patients with alcohol dependence in our study, which is in line with data from the World Health Organization (WHO) (2014) [22]. According to the WHO, the 2010 per capita average alcohol consumption among male and female alcohol drinkers in Poland was 31.5 L and 14.0 L pure alcohol, respectively [22].

Living in a dysfunctional household with biological parent(s) with alcohol abuse/dependence is associated with a variety of genetic and environmental factors that influence lifetime risk of alcohol dependence. Epidemiological studies have found that first-degree relatives of individuals with alcohol dependence are two to seven times more likely than people with non-alcoholic relatives to develop alcohol dependence during their lifetime [6]. However, individuals with alcohol dependent first-degree relative are undoubtedly not destined to develop alcohol dependence at some point in their life. Indeed, in our sample, the majority of the male patients (61.2%) and over half (50.9%) of the female patients reported living with parents without alcohol abuse/dependence.

Notably, the age at onset of drinking was found to be an independent risk factor for the development of alcohol dependence. Among individuals with and without a first-degree alcohol-dependent relative, the likelihood of lifetime alcohol dependence was shown to decrease with an increasing age of drinking onset [20,23]. Here, we assessed age at alcohol initiation, defined as age at the first intake of any amount of alcohol. We suggest that age at alcohol initiation may serve as a marker of household dysfunction, including poor parental management. In a previous study by Berent et al. (2018), emotional neglect during childhood and adolescence was reported by 44% of adult female patients and 35% of adult male patients with alcohol dependence [13]. Undoubtedly, not all children intoxicated with alcohol during their first years of life grow up in households with alcohol abuse/dependence parent(s). Indeed, an analysis of archival medical records of Polish pediatric units between the years 2000 and 2011 revealed that about 10% of mothers and over 20% of fathers of children admitted due to alcohol intoxication were diagnosed with alcohol dependence [11]. In the present study, male patients were significantly more likely to report parents without vs. with alcohol abuse/dependence, compared to females. However, the mean age at first alcohol intake was significantly younger in males than in females in the overall study group. Moreover, males who reported living with parents with a history of alcohol abuse/dependence were younger than females at first alcohol intake when compared to the same subgroup with parental history of alcohol abuse/dependence. This finding suggests the at least partial modifiability of the role of genetics in the development of alcohol dependence. The environment and personality traits may either enhance or reduce the impact of genetics on the development of alcohol dependence. As reported by Guo et al. (2001), a strong bonding to school, close parental monitoring of children, clearly defined family rules for behavior, appropriate parental rewards for good behavior, a high level of refusal skills, and a strong belief in moral order are shown to predict a lower risk for alcohol abuse and dependence at age 21 [24]. We suggest that sex differences in the age at first alcohol intake may be due to differences in temperament, novelty-seeking, and role assignment in dysfunctional households. In particular, compared to boys, girls are more frequently caregivers who provide support for parents and siblings [5,25,26,27]. There is a global need for systemic interventions to help alcohol abusing/dependent parents to carry out their parental responsibilities. For example, interventions may provide social support, resources, and education for parents, as well as develop sources of social support outside the family and provide opportunities for the socialization of children and adolescents [2,28].

In our study, the youngest reported age at alcohol initiation was 10 among females and 3 among males with alcohol dependence. The youngest child with alcohol intoxication admitted to a Polish pediatric unit was a 1.6-year-old in a study by Kamińska et al. (2018) [11] and a 2.4-year-old in a study by Pawłowska-Kamieniak et al. (2011) [29]. Epidemiological studies have found that individuals who start drinking at age 14 or younger are approximately four times more likely to develop alcohol dependence, compared to those who begin drinking at age 20 or older [20]. In our study, age at alcohol initiation ≤ 14 years was reported by 10 (17.54%) female patients with alcohol dependence and 82 (34.60%) male patients with alcohol dependence. Further, the median age at alcohol initiation was 16±3.81 years (Me ± SD) in males and 17±5.98 years (Me ± SD) in females. Male patients in our sample were younger at first alcohol intake than female patients with alcohol dependence. In further subgroup analyses, we found that male patients without parental history of alcohol abuse/dependence, maternal only history of alcohol abuse/dependence, and paternal only history of alcohol abuse/dependence were younger at first alcohol intake than their female counterparts, although these differences did not reach statistical significance. However, there was a significant sex difference between subgroups raised by both parents with alcohol abuse/dependence, such that males were significantly younger at alcohol initiation than females. Among female patients, there were no differences between subgroups in the age at alcohol initiation. The median age at alcohol initiation was 17 years among females raised by parents without alcohol abuse/dependence or alcohol abusing/dependent father. The median age was slightly younger (16 years) when females were raised by an alcohol-abusing/dependent mother, and slightly older (18 years) when raised in a household in which both parents have alcohol abuse/dependence. Age at first alcohol intake differed significantly among subgroups of male patients with alcohol dependence, such that male patients raised by parents without alcohol abuse/dependence were older at first alcohol intake than male patients who grew up with both parents with alcohol abuse/dependence.

### Limitations

The present study consisted of adult patients recruited from inpatient treatment programs for alcohol dependence and may, therefore, not be representative of all patients with alcohol dependence.

The cross-sectional design of our study required adult patients with alcohol dependence to retrospectively report alcohol abuse/dependence among their parent(s) and the age at first alcohol intake.

Only patients who reported none, one, or both parents with alcohol abuse/dependence entered our analysis for association with age at first alcohol intake. Studies involving larger samples where associations with the history of alcohol abuse/dependence of other household members are analyzed are still required. A strength of our study is that self-reporting on family alcohol abuse/dependence shows good to excellent reliability, with high kappa values for parents (kappa of 0.72 for fathers’ and 1.00 for mothers’ history of alcohol abuse/dependence) [20].

## 5. Conclusions

This study provides new insights into the impact of parental history of alcohol abuse/dependence on the age at alcohol initiation, as retrospectively self-reported by adult patients with alcohol dependence. We found that males are more prone to drinking alcohol for the first time at a younger age than females, and that this pattern is more pronounced among those with alcohol abuse/dependence in both parents. Further studies that include a larger number of female patients are required to assess differences in age at alcohol initiation between males and females with and without a history of alcohol abuse/dependence in one or both parents.

## Figures and Tables

**Table 1:** Socio-demographics and descriptive characteristics of study patients with alcohol dependence (*n* = 294).

Characteristic		*n* = 294	Males (*n* = 237)	Females (*n* = 57)
Sex *n* (%)			237 (80.6)	57 (19.4)
Age at survey	M (SD)	43.85 (10.96)	43.54 (10.81)	45.14 (11.56)
	Me (IQR)	42.0 (16.25)	42.0 (15.5)	45.0 (20.5)
Place of living *n* (%)	Village	43 (14.6)	37 (15.6)	6 (10.5)
	Urban area	251 (85.4)	200 (84.4)	51 (89.5)
Educational level *n* (%)	Elementary	58 (19.7)	51 (21.5)	7 (12.3)
	Vocational	84 (28.6)	69 (29.1)	15 (26.3)
	High School	106 (36.1)	83 (35.0)	23 (40.4)
	University degree	46 (15.6)	34 (14.3)	12 (21.1)
Employment status *n* (%)	Employed	107 (36.4)	87 (36.7)	20 (35.1)
	Unemployed	150 (51.0)	123 (51.9)	27 (47.4)
	Retired/sickness leave	37 (12.6)	27 (11.4)	10 (17.5)
Marital status *n* (%)	Never married	127 (43.2)	110 (46.4)	17 (13.4)
	Married	64 (21.8)	51 (21.5)	13 (22.8)
	Divorced/Separated	88 (30.0)	69 (29.1)	19 (33.4)
	Widowed	15 (5.0)	7 (3.0)	8 (14.0)
Age at first alcohol intake	M (SD)	16.14 (4.40)	15.69 (3.81)	18.00 (5.98)
	Me (IQR)	16.0 (2.0)	16.0 (3.5)	17.0 (3.0)
AUDIT score	M (SD)	26.76 (7.63)	27.70 (7.27)	22.84 (7.89)
	Me (IQR)	28.0 (12.0)	28.0 (11.5)	22.0 (12.5)
History of parental alcohol abuse/dependence *n* (%)	None	174 (59.18)	145 (61.2)	29 (50.9)
	Mother	20 (6.80)	17 (7.2)	3 (5.3)
	Father	84 (28.57)	67 (28.3)	17 (29.8)
	both	16 (5.44)	8 (3.4)	8 (14.0)

AUDIT—Alcohol Use Disorders Identification test; M (SD)—Mean (Standard deviation); Me—median; IQR—interquartile range; Z—Mann–Whitney test; *p*—level of statistical significance; r—effect size; V—Cramer’s V.

**Table 2:** Age at first alcohol intake and history of parental alcohol abuse/dependence by male and female patients with alcohol dependence (*n* = 294).

	**Parents without History of Alcohol Abuse/Dependence**								
	**Female Patients (*n* = 29)**			**Male Patients (*n* = 145)**					
	**M_range_**	**Me**	**IQR**	**M_range_**	**Me**	**IQR**	**Z**	***p***	**r**
Age at first alcohol intake	95.86	17.0	4.0	85.83	16.0	4.0	−0.99	0.323	0.08
	**Alcohol Abuse/Dependent Mother**								
	**Female Patients (*n* = 3)**			**Male Patients (*n* = 17)**					
	**M_range_**	**Me**	**IQR**	**M_range_**	**Me**	**IQR**	**Z**	***p***	**r**
Age at first alcohol intake	12.33	16.0	3.0	10.18	15.0	3.5	0.56	0.616	0.13
	**Alcohol Abuse/Dependent Father**								
	**Male Patients (*n* = 17)**			**Female Patients (*n* = 67)**					
	**M_range_**	**Me**	**IQR**	**M_range_**	**Me**	**IQR**	**Z**	***p***	**r**
Age at first alcohol intake	52.03	17.0	3.5	40.08	15.0	4.0	−1.82	0.069	0.20
	**Both Parents with Alcohol Abuse/Dependence**								
	**Female Patients (*n* = 8)**			**Male Patients (*n* = 8)**					
	**M_range_**	**Me**	**IQR**	**M_range_**	**Me**	**IQR**	**Z**	***p***	**r**
Age at first alcohol intake	12.00	18.0	17.25	5.00	12.0	6.25	−2.97	0.002	0.74

M_range_—Medium range; Me—median; IQR—interquartile range; Z—Mann–Whitney test; *p*—level of statistical significance; r—effect size.

**Table 3:** Comparison of age at first alcohol intake in female and male patients with alcohol dependence reporting a parental history of alcohol abuse/dependence.

	**Female Patients (*n* = 57)**														
	**None**			**Mother Only**			**Father Only**			**Both Parents**					
	**M_range_**	**Me**	**IQR**	**M_range_**	**Me**	**IQR**	**M_range_**	**Me**	**IQR**	**M_range_**	**Me**	**IQR**	**H**	***p***	**η^2^**
Age at first alcohol intake	27.45	17.0	4.0	21.67	16.0	3.0	28.62	17.0	3.5	38.19	18.0	17.25	3.37	0.338	0.06
	**Male Patients (*n* = 237)**														
	**None of Parents**			**Mother Only**			**Father Only**			**Both Parents**					
	**M_range_**	**Me**	**IQR**	**M_range_**	**Me**	**IQR**	**M_range_**	**Me**	**IQR**	**M_range_**	**Me**	**IQR**	**H**	***p***	**η^2^**
Age at first alcohol intake	126.88	16.0	4.0	97.76	15.0	3.5	114.49	15.0	4.0	59.00	12.0	6.25	10.10	0.018 *	0.04

M_range_—Medium range; Me—median; IQR—interquartile range; H—Kruskal–Wallis test; *p*—level of statistical significance; η^2^—effect size. * Post hoc analysis: male patients with alcohol dependence reporting both parents with alcohol abuse/dependence were younger at first alcohol intake than male patients with alcohol dependence reporting parents without alcohol abuse/dependence (*p* = 0.036).

## Data Availability

The data sets used and analyzed in the current study are available from the corresponding author upon reasonable request.

## Supplementary Materials

The following are available online at https://www.mdpi.com/article/10.3390/healthcare9070841/s1, Supplementary Table S1. Descriptive statistics of current age, age at first alcohol intake, and AUDIT scores among male (*n* = 237) and female (*n* = 57) patients with alcohol dependence.
