# Supplementary material for: Does Parental Alcohol Use Influence Children’s Age at First Alcohol Intake? A Retrospective Study of Patients with Alcohol Dependence
Source: Healthcare (Basel). 2021 Jul 3;9(7):841. doi: 10.3390/healthcare9070841 (PMC8303690; doi:10.3390/healthcare9070841)
Supplement: Supplementary file 1 [file healthcare-09-00841-s001.zip › healthcare-1260859-supplementary.pdf]

Supplementary Material

Table S1. Descriptive statistics of current age, age at first alcohol intake, and AUDIT scores among male (n = 237) and female (n = 57) patients with alcohol dependence.

	**M**	**Me**	**SD**	**Sk**	**Kurt**	**Min**	**Max**	**D**	***p***
**Females**									
Current age	45.14	45.0	11.56	-0.04	-1.24	26	65	0.09	0.200
Age at first alcohol intake	18.00	17.0	5.98	2.90	9.24	10	43	0.31	<0.001
AUDIT score	22.84	22.0	7.89	0.03	-0.73	6	37	0.09	0.200
**Males**									
Current age	43.54	42.0	10.81	0.46	-0.53	22	73	0.08	<0.001
Age at first alcohol intake	15.69	16.0	3.81	0.42	4.53	3	34	0.15	<0.001
AUDIT score	27.70	28.0	7.27	-0.36	-0.51	4	40	0.07	0.005

M—Mean; Me—Median; SD—Standard Deviation; Sk—skewness; Kurt—kurtosis; Min—minimal value; Max—maximal value; D—Lilliefors corrected Kolmogorov-Smirnov test value; *p*—level of statistical significance.
